# Relationship of early brain growth pattern measured by ultrasound with neurological outcome at two years of age in very low birth weight infants

**DOI:** 10.1007/s00431-023-05170-2

**Published:** 2023-09-08

**Authors:** Estefanía Ruiz-González, Simón P. Lubián-López, Natalia Jiménez Luque, Antonio Segado-Arenas, Manuel Lubián-Gutiérrez, Yolanda Marín Almagro, Pamela Zafra-Rodríguez, Paula Méndez-Abad, Isabel Benavente-Fernández

**Affiliations:** 1 Department of Paediatrics, Neonatology Section, Puerta del Mar University Hospital, Cádiz, Spain; 2 Biomedical Research and Innovation Institute of Cádiz (INiBICA) Research Unit, Puerta del Mar University Hospital, Cádiz, Spain; 3 Paediatrics Area, Department of Mother and Child Health and Radiology, Medical School, University of Cádiz, C/Doctor Marañon, 3, , Cádiz, Spain

**Keywords:** Preterm, Very low birth weight infants, Brain volume, Brain growth, Ultrasonography, Neurodevelopment

## Abstract

The purpose of this study is to define the impact of early brain growth trajectory in very low birth weight infants (VLBWI) on neurological prognosis at 2 years, assessed using sequential ultrasound (US) scans. This is a prospective cohort study with consecutive inclusion of VLBWI ≤ 32 weeks gestational age and ≤ 1500 g at birth. Total brain volume (TBV) was assessed using sequential 3D-US from birth to discharge. Prognosis at 2 years (corrected age) was assessed using the Bayley Scales of Infant and Toddler Development Third Edition. TBV showed slower growth with postmenstrual age (PMA) in those VLBWI who had an adverse cognitive prognosis compared to those with good cognitive prognosis (mean difference in TBV between prognostic groups from 4.56 cm^3^ at 28 weeks to 42.58 cm^3^ at 43 weeks) as well as in those with adverse language prognosis (mean difference in TBV from 2.21 cm^3^ at 28 weeks to 26.98 cm^3^ at 43 weeks) although other variables showed more impact than TBV on language prognosis (gestational age at birth, brain injury at term, and socioeconomic status). No association was found between TBV and motor prognosis. Brain growth rate was also significantly higher in those VLBWI who presented good cognitive scores (18.78 + (0.33 × (PMA-33)) cm^3^/week) compared to those with adverse cognitive outcome (13.73 + (0.64 × (PMA-33)) cm^3^/week).

*  Conclusion*: Early altered brain growth is associated with poor cognitive prognosis at 2 years of age. Using sequential US monitoring, we can detect early brain growth deviation in patients who will have adverse cognitive outcomes.

**Table:** 

**What is known:** *• The prediction of neurodevelopmental outcome of VLBWI is mostly based on the presence of brain injury in US and structural magnetic resonance imaging (MRI) at term. * *• Some studies have related brain volume measured on MRI at term with neurodevelopment outcome.*	
**What is new:** *• VLBWI with adverse cognitive prognosis at two years of age present smaller brain volumes detectable by sequential US during NICU admission.* *• Brain volume can be estimated from 2D and 3D US and has prognostic value in VLBWI.*

**Supplementary information:**

The online version contains supplementary material available at 10.1007/s00431-023-05170-2.

## Introduction

Very low birth weight infants (VLBWI) are a high-risk population exposed to high-morbidity rates and a wide spectrum of long-term neurodevelopmental abnormalities. Although advances in perinatal medicine have led to an increase in the survival rates at extreme gestational ages [1–5], the long-term neurologic outcome of these patients remains a matter of concern due to high rates of neurodevelopmental disorders including intellectual deficits, behavioral disorders, cerebral palsy, and epilepsy [3, 4, 6–10]. These sequelae of prematurity have a great impact on the child’s future health, with notable family and social impact.

Brain imaging using ultrasound (US) and magnetic resonance imaging (MRI) is a valuable tool during neonatal admission to diagnose brain injury, assisting the clinician to predict the long-term outcome of preterm infants. The increasing research interest in the developing brain together with the technological improvement of these tools has led to a better understanding of the impact of prematurity on the immature brain. MRI is considered the gold standard but cannot rival US for its role in sequential and incubator-based neurological assessment in the preterm infant. Aside from brain injury, US has the potential to estimate brain volumes as reliably as MRI [11] allowing the study of early brain growth patterns. Moreover, we have previously demonstrated that those preterm infants born at lower gestational age and exposed to an increasing number of comorbidities have a deviated pattern of brain growth [12]. However, our study was previously based on the results of term-equivalent MRI while our aim in this study is to identify the pattern of early brain growth in the preterm infants in relation to the 2-year neurodevelopmental outcome.

## Materials and methods

### Study population

This longitudinal study included VLBWI admitted to the Hospital Puerta del Mar, Cádiz, Spain, from May 2018 to January 2021. We consecutively enrolled those VLBWI with a birth weight equal or less than 1500 g and/or a gestational age at birth equal or less than 32 weeks. The exclusion criteria were defined as the presence of congenital or chromosomal anomalies, metabolic disorders, and central nervous system infections. We also excluded those preterm infants who presented posthemorrhagic ventricular dilatation or died. This study was approved by the Research and Ethics Committee, and all parents or guardians of the participants provided informed consent.

Perinatal and postnatal variables were prospectively collected (see Table S1 in Supplemental material for a detailed definition of the clinical variables).

### 2D and 3D brain US

Weekly 2D and 3D brains US were carried out while the included patients were admitted to the neonatal intensive care unit (NICU), with the infant lying supine and their head turned to the right. Volume acquisition was carried out using the 3D option of the 3D/4D Voluson S8 BT18 (General Electric Healthcare, Buckinghamshire, United Kingdom) as explained elsewhere [11].

TBV was measured by manual tracing the brain contour on 6 slices at 30 degrees rotation on the vertical axis using VOCAL (Virtual Organ Computer-Aided Analysis) feature of the 4D View software (version 17.0; GE Healthcare). This technique provides a reliable measure of TBV as previously published by our group [11].

### Brain MRI

At term-corrected age, all patients underwent a cranial MRI. MRI scans were performed using 1.5 T scanner Magneton Symphony (Siemens Health Care, Erlangen, Germany) located in the radiology unit. T1-weighted images were obtained using a three-dimensional spoiled gradient [repetition time 1660 (RT)/echo time 5.16(ET)] and transverse T2-weighted turbo spin-echo imaging (4180.00/98.00).

Term-MRI scans were evaluated using the scale published by Kidokoro et al. [13], which separately grades the development and injury of the cortical and deep gray matter, white matter and cerebellum. Those with a score of less than 8 points were considered to have normal/mild abnormalities at term-MRI, while those with a score equal or greater than 8 points were classified as having moderate/severe abnormalities at term-MRI.

### Assessment at two years of age

All the included patients were reviewed after discharge as part of the neonatal neurology follow-up program. Assessments at two years of corrected age were performed using the Bayley Scales of Infant and Toddler Development, Third Edition (Bayley-III). The scores obtained on the cognitive, motor, and language scales are standardized with a mean of 100 and a standard deviation of 15. We considered both the quantitative data and further dichotomized the scores considering a good neurodevelopmental outcome if the score was greater than or equal to 85 and adverse outcome if they scored under 85 for each scale.

### Statistical analysis

Clinical characteristics and demographic variables were described as frequency and percentage if categorical, or mean and standard deviation (sd), or median and interquartile range [IQR] according to their distribution. Bivariate analysis was performed using Pearsonʼs chi-squared test or Fisherʼs exact test for categorical data and Studentʼs *t*-test or Mann–Whitney *U* test for continuous variables after testing for normality. Multilevel linear regression models were used to study the relationship between Bayley-III scores, TBV, and clinical variables accounting for repeated measurements and time. The included variables were selected based on the theoretical background, and a backward stepwise approach was performed to exclude the non-significant variables if not considered to be variables for which an adjustment was needed.

Statistical analysis was conducted using Stata 16.0 (Stata Statistical Software: Release 16. College Station, TX: StataCorp LP). A result was considered statistically significant at *p* < 0.05.

## Results

### Clinical characteristics of the studied population and 2-year neurodevelopmental outcomes

For this study, we included those VLBWI who were admitted to the NICU at Puerta del Mar Hospital from May 2018 to January 2021, including a total population of 163 patients. Nineteen (11.7%) of these patients died during the neonatal period. Six (3.7%) patients were excluded: one patient for congenital cytomegalovirus infection (CMV), a patient with Down syndrome, and four patients for developing posthemorrhagic ventricular dilatation (PHVD). Of the remaining 138 patients, our final sample size included 105 (76.1%) that completed the assessment at 2 years of corrected age. These patients had a combined total of 719 brain US during their stay in the NICU (see Fig. S1 in Supplemental material).

Our population had a mean gestational age at birth of 29.3 (± 2.3) weeks and mean birth weight of 1168.6 (± 363.1) grams. Seventeen patients (16.19%) were small for gestational age (SGA). A detailed description of the perinatal variables, socioeconomic status, and comorbidities related to the 2-year neurodevelopmental outcome is shown in Table S2 in Supplemental material.

#### Motor outcome

Those with an adverse motor outcome had a lower proportion of exposure to prenatal steroids compared to those with a good motor outcome (7/13 (53.85%) vs. 75/88 (85.23%); *p* = 0.015), a higher proportion of severe retinopathy of prematurity (ROP) (4/13 (30.77%) vs. 3/91 (3.3%); *p* = 0.004), moderate/severe bronchopulmonary dysplasia (BPD) (6/13 (46.15%) vs. 13/90 (14.44%); *p* = 0.014), intraventricular hemorrhage (IVH) grade 3 (3/13 (23.08%) vs. 3/92 (3.26%); *p* = 0.024), moderate/severe white matter injury (WMI) (2/13 (15.38%) vs. 0/92 (0%); *p* = 0.014), higher scores on the Kidokoro scale (2 [0–10] vs. 0 [0–2]; *p* = 0.03), and a greater number of comorbidities (1 [0–3] vs. 0 [0–1]; *p* = 0.006), respectively (see Table S2 in Supplemental material).

#### Cognitive outcome

The proportion of multiple births 7/9 (77.78%) was higher in those with an adverse cognitive outcome when compared to those with a good cognitive outcome (34/96 (35.42%); *p* = 0.026). No other differences were found in the baseline characteristics of the studied population related to cognitive outcome (see Table S2 in Supplemental material).

#### Language outcome

Sex was associated with language scores at 2 years with females having better outcomes: 47 females/83 (56.63%) had good vs. 6 females/22 (27.27%) with an adverse language outcome; *p* = 0.001. Adverse language outcome was related to severe ROP (4/22 (18.88%) vs. 3/82 (3.66%); *p* = 0.035) and moderate/severe WMI (2/22 (9.09%) vs. 0/83 (0%); *p* = 0.042) (see Table S2 in Supplemental material).

### Total brain volume (TBV) during early postnatal life related to 2-year neurodevelopmental outcome

We studied the association of sequential measurements of TBV, gestational age (GA) at birth, and PMA at the time of US, with the 2-year neurodevelopmental outcome (see Table 1). TBV was related to cognitive and language outcome, while no association was found with motor outcome.

**Table 1:** Total brain volume (TBV), GA at birth, and PMA related to 2-year neurodevelopmental outcomes

	**Motor score**		**Cognitive score**		**Language score**	
	**Coef *β***	***p***	**Coef *β***	***p***	**Coef *β***	***p***
**Const**	67.207	**0.0001**	75.049	**0.0001**	72.534	**0.0001**
**TBV**	0.004	0.787	0.035	**0.01**	0.051	**0.003**
**Gestational age (GA) at birth**	0.987	**0.0001**	1.001	**0.0001**	1.411	**0.0001**
**Postmenstrual age (PMA)**	0.006	0.979	−0.359	0.13	−0.871	**0.003**
	No. of obs = 707/No. of groups = 105 Wald chi^2^ = 23.95/*p* model = 0.00001		No. of obs = 707/No. of groups = 105 Wald chi^2^ = 36.93/*p* model = 0.00001		No. of obs = 707/No. of groups = 105 Wald chi^2^ = 39.2/*p* model = 0.00001	

Values in bold indicate statistical significance

**Fig. 1:**
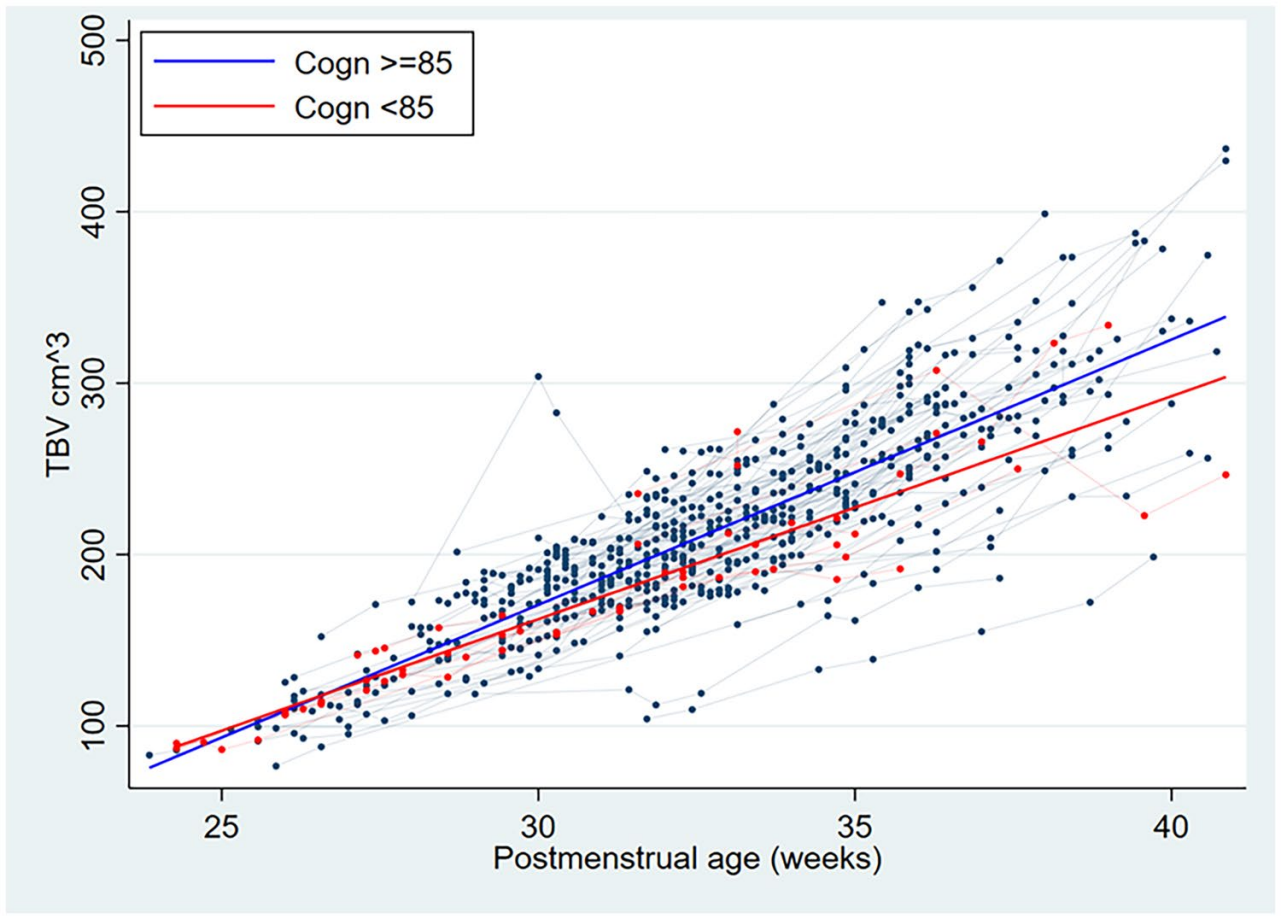
Total brain volume (TBV) during early postnatal life related to cognitive outcome

TBV showed a slower increased related to PMA in those VLBWI who had an adverse cognitive outcome, with mean TBV differences between both outcome groups being significant from 28 weeks PMA onwards and ranging from 4.56 cm^3^ at 28 weeks PMA to 42.58 cm^3^ at 43 weeks PMA (see Table 2 and Fig. 1).

**Table 2:** TBV differences in VLBWI with good versus adverse cognitive outcome by PMA

PMA (weeks)	Good cognitive outcome	Adverse cognitive outcome	Absolute Diff	*p*
24	88.82 (± 3.18)	94.75 (± 2.63)	−5.93	0.99
25	106.63 (± 3.56)	109.47 (± 2.94)	−2.84	0.94
26	118.11 (± 4.16)	118.95 (± 3.44)	−0.84	0.76
27	133.15 (± 3.96)	131.38 (± 3.27)	1.77	0.056
28	149.19 (± 4.33)	144.63 (± 3.58)	4.56	**0.0001**
29	164.38 (± 3.86)	157.19 (± 3.19)	7.19	**0.00001**
30	177.99 (± 4.14)	168.44 (± 3.43)	9.56	**0.00001**
31	193.49 (± 4.39)	181.24 (± 3.62)	12.25	**0.00001**
32	207.66 (± 4.29)	192.95 (± 3.53)	14.71	**0.00001**
33	223.45 (± 4.51)	205.99 (± 3.73)	17.45	**0.00001**
34	238.76 (± 4.68)	218.65 (± 3.87)	20.11	**0.00001**
35	253.81 (± 4.34)	231.08 (± 3.59)	22.72	**0.00001**
36	265.99 (± 3.63)	241.15 (± 2.99)	24.84	**0.00001**
37	281.84 (± 4.43)	254.25 (± 3.66)	27.59	**0.00001**
38	296.59 (± 3.91)	266.43 (± 3.23)	30.15	**0.00001**
39	311.31 (± 4.69)	278.6 (± 3.87)	32.71	**0.00001**
40	328.14 (± 5.03)	292.5 (± 4.15)	35.63	**0.00001**
41	337.39 (± 3.96)	300.15 (± 3.27)	37.24	**0.00001**
42	356.79 (± 4.25)	316.18 (± 3.51)	40.61	**0.00001**
43	368.11 (± 3.24)	325.54 (± 2.68)	42.58	**0.00001**
Overall	219.9 (54.34)	203.06 (± 49.04)	16.84	**0.00001**

Values in bold indicate statistical significance

**Fig. 2:**
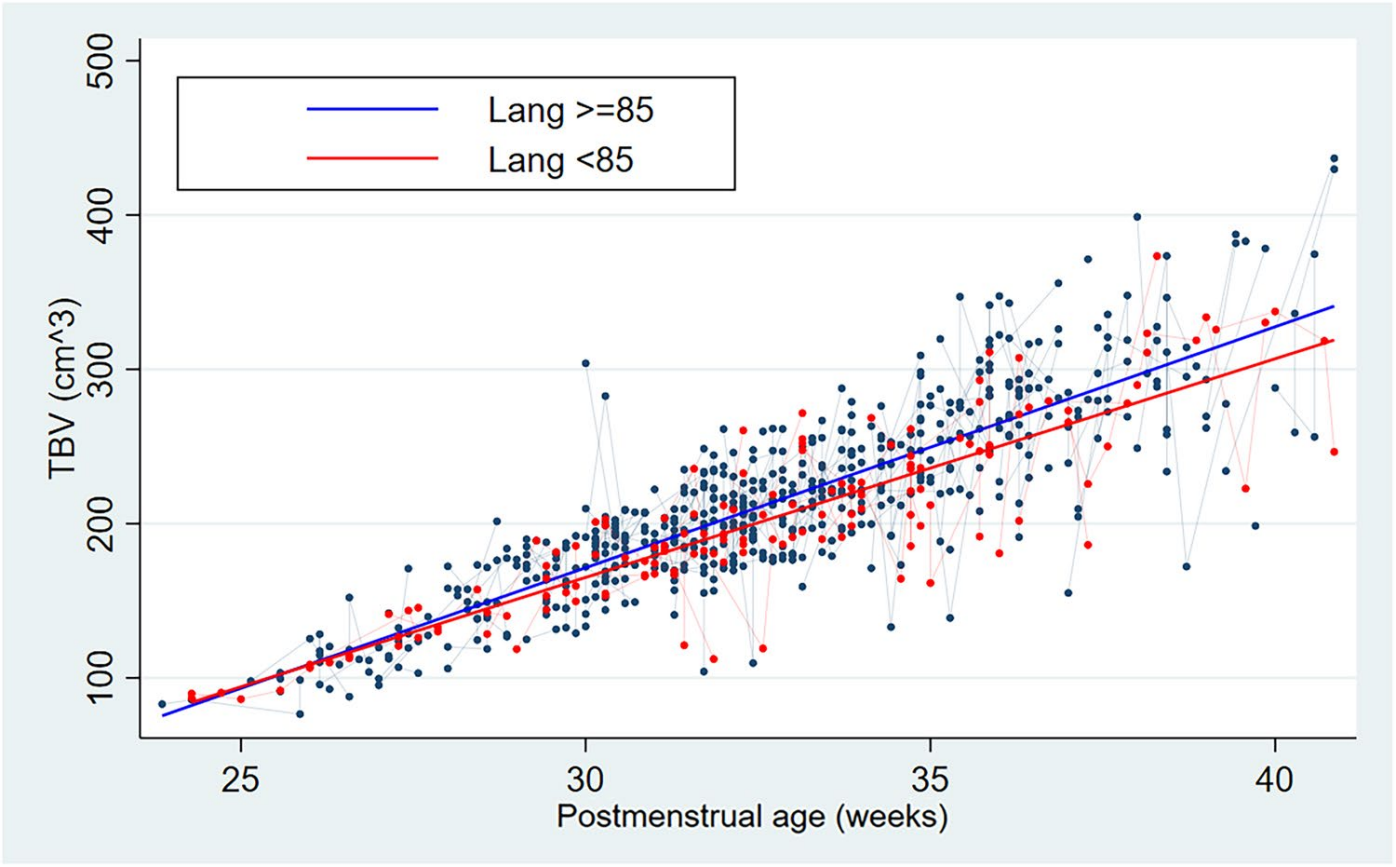
Total brain volume (TBV) during early postnatal life related to language outcome

Similarly, TBV showed a slower increase related to PMA in those VLBWI who had an adverse language outcome, with mean TBV differences between both outcome groups being significant from 28 weeks PMA onwards and ranging from 2.21 cm^3^ at 28 weeks PMA to 26.98 cm^3^ at 43 weeks PMA (see Table 3; Fig. 2).

**Table 3:** TBV differences in VLBWI with good versus adverse language outcome by PMA

PMA (weeks)	Good language outcome	Adverse language outcome	Absolute Diff	*p*
24	86.45 (± 3.27)	91.08 (± 2.91)	−4.62	0.96
25	104.76 (± 3.66)	107.37 (± 3.25)	−2.61	0.91
26	116.56 (± 4.27)	117.87 (± 3.8)	−1.31	0.84
27	132.02 (± 4.07)	131.63 (± 3.62)	0.39	0.37
28	148.51 (± 4.45)	146.3 (± 3.96)	2.21	**0.03**
29	164.12 (± 3.97)	160.2 (± 3.53)	3.92	**0.00001**
30	178.11 (± 4.26)	172.65 (± 3.79)	5.46	**0.00001**
31	194.04 (± 4.51)	186.82 (± 4.01)	7.22	**0.00001**
32	208.61 (± 4.39)	199.79 (± 3.9)	8.82	**0.00001**
33	224.84 (± 4.63)	214.23 (± 4.12)	10.61	**0.00001**
34	240.57 (± 4.81)	228.4 (± 4.28)	12.34	**0.00001**
35	256.04 (± 4.46)	241.99 (± 3.97)	14.04	**0.00001**
36	268.56 (± 3.73)	253.14 (± 3.32)	15.42	**0.00001**
37	284.85 (± 4.56)	267.64 (± 4.06)	17.21	**0.00001**
38	300.01 (± 4.01)	281.13 (± 3.57)	18.88	**0.00001**
39	315.14 (± 4.82)	294.59 (± 4.29)	20.55	**0.00001**
40	332.44 (± 5.17)	309.99 (± 4.59)	22.45	**0.00001**
41	341.94 (± 4.07)	318.44 (± 3.62)	23.5	**0.00001**
42	361.89 (± 4.36)	336.19 (± 3.88)	25.69	**0.00001**
43	373.53 (± 3.33)	346.55 (± 2.97)	26.98	**0.0002**
Overall	221.18 (± 60.99)	210.98 (± 54.28)	10.21	**0.0007**

Values in bold indicate statistical significance

### Association of perinatal factors, maternal level of education, comorbidities, and brain injury with the prognostic scores

#### Motor outcome

Motor outcome at 2 years was related to GA at birth (*β* coef = 0.52; *p* = 0.008), sex (*β* coef (female) = 2.23; *p* = 0.025), being SGA (*β* coef = -4.4; *p* = 0.0001), and moderate/severe findings in term-equivalent MRI (*β* coef = −14.87; *p* = 0.0001). We did not find a statistically significant association of brain volumes during NICU admission and socioeconomic status (SES) on motor scores (see Table 4).

**Table 4:** Association of perinatal factors, SES, comorbidities, and brain injury with prognostic outcome

		**Motor score**		**Cognitive score**		**Language score**	
**Variables**		**Coef**	***p***	**Coef**	***p***	**Coef**	***p***
**Const**		85.47	**0.0001**	86.54	**0.0001**	81.14	**0.0001**
**Gestational age (weeks)**		0.52	**0.008**	0.56	**0.005**	0.86	**0.0001**
**Sex (F)**		2.23	**0.025**	2.13	**0.033**	1.37	0.269
**SGA**		−4.4	**0.0001**	−2.67	**0.028**	−1.6	0.29
**TBV (cm**^**3**^**)**		−0.01	0.709	0.02	**0.037**	0.01	0.438
**Term MRI (Kidokoro mod/sev)**		−14.87	**0.0001**	−12.87	**0.0001**	−14.95	**0.0001**
**Maternal level of education**	**Low**	−2.76	0.067	−5.7	**0.0001**	−11.85	**0.0001**
	**Medium**	−0.18	0.907	−3.01	0.061	−10.51	**0.0001**
	**High**	Ref	Ref	Ref	Ref	Ref	Ref
		No. of obs = 629/No. of groups = 17 Wald chi^2^ = 212.36/*p* model = 0.00001		No. of obs = 629/No. of groups = 17 Wald chi^2^ = 183.37/*p* model = 0.00001		No. of obs = 629/No. of groups = 17 Wald chi^2^ = 177.77/*p* model = 0.00001	

Values in bold indicate statistical significance

*SGA* Small for Gestational Age, *TBV* Total Brain Volume, *MRI* Magnetic Resonance Imaging

#### Cognitive outcome

Cognitive outcome was associated to GA at birth (*β* coef = 0.56; *p* = 0.005), sex (*β* coef (female) = 2.13; *p* = 0.033), SES (low SES group *β* coef = −5.7; *p* = 0.0001), being SGA (*β* coef = −2.67; *p* = 0.028), moderate/severe abnormalities on term-MRI (*β* coef = −12.87; *p* = 0.0001), and TBV (*β* coef = 0.02; *p* = 0.037) (see Table 4).

#### Language outcome

Language outcome at 2 years was associated with GA at birth (*β* coef = 0.86; *p* = 0.0001), SES (low SES group *β* coef = −11.85; *p* = 0.0001; medium SES group *β* coef = −10.51; *p* = 0.0001), moderate/severe abnormalities at term MRI (*β* coef = −14.26; *p* = 0.0001) while brain volume in early postnatal life was not related to language scores (see Table 4).

### Brain growth rate related to cognitive outcome

**Fig. 3:**
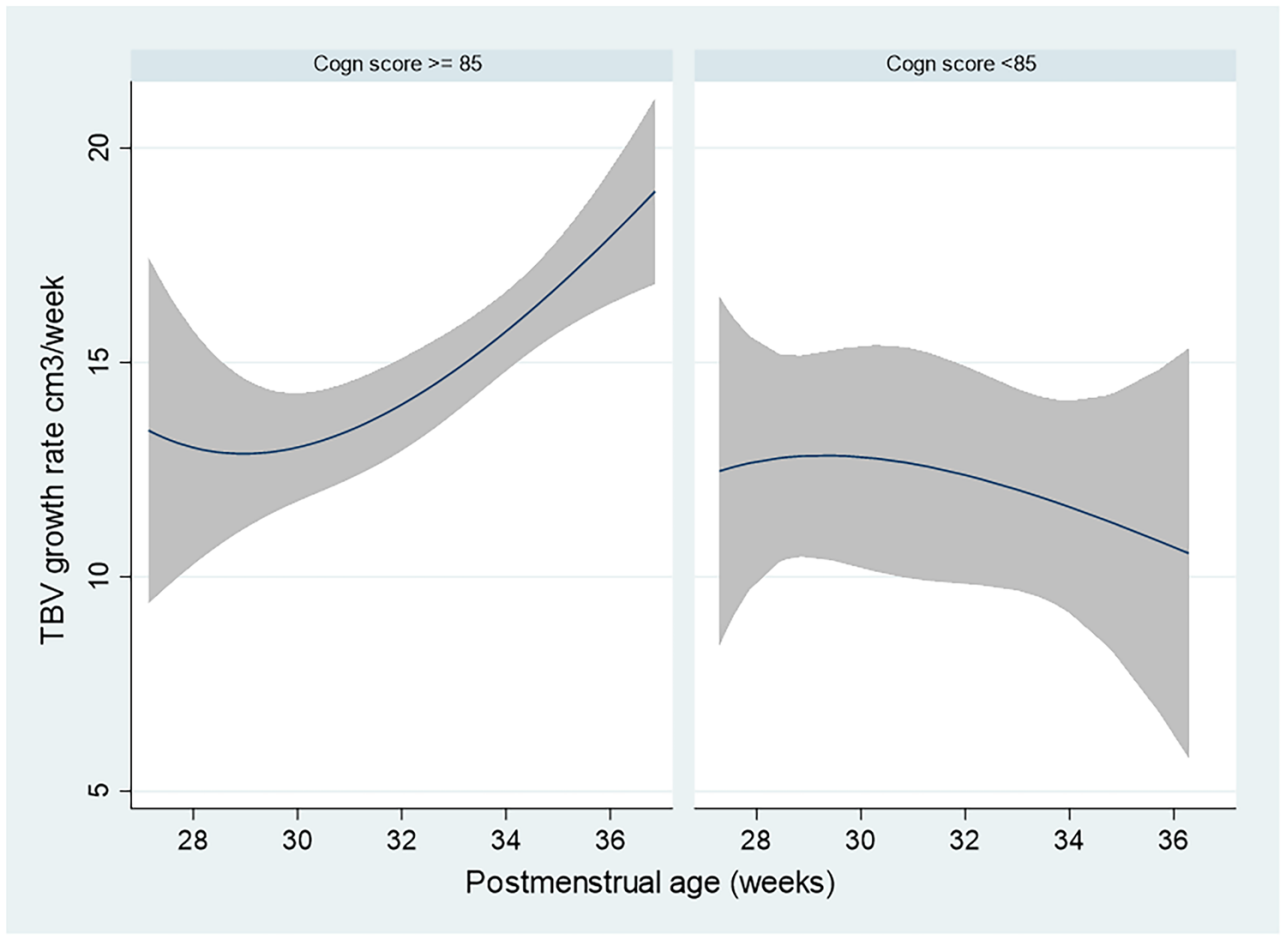
Brain growth rate during early postnatal life related to cognitive outcome

As TBV was more consistently related to cognitive outcome, we explored brain growth rate related to the 2-year cognitive outcome. We calculated brain growth rate as the difference in TBV between two consecutive ultrasound scans divided by time (cm^3^/week). In those VLBWI with good cognitive outcome, brain growth rate was as follows: TBV growth rate (cm^3^/week) = 18.78 + (0.33 × (PMA-33)) while in those with an adverse cognitive outcome, it was adjusted to the following equation: TBV growth rate = 13.73 + (0.64 × (PMA-33)) (see Fig. 3 and Table S3 in Supplemental material).

### TBV during early postnatal life in VLBWI with normal cognitive outcome related to PMA as a reference for clinical use

**Fig. 4:**
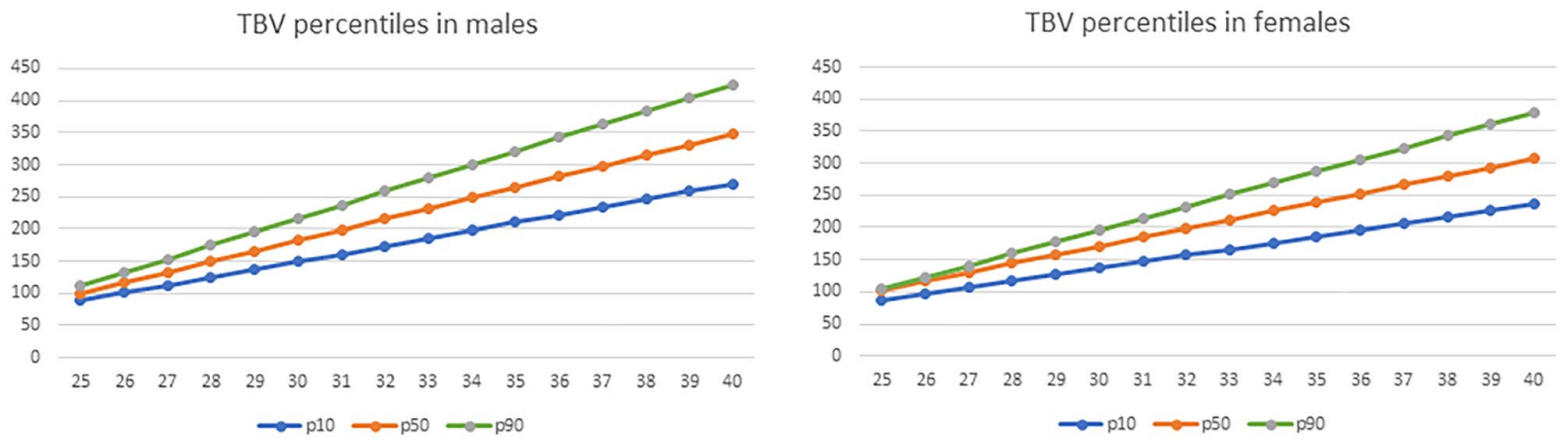
TBV percentiles (cm^3^) by postmenstrual age in preterm infants with normal 2-year cognitive outcome

To facilitate establishing routine TBV monitoring during NICU admission of VLBWI, we estimated the TBV percentiles by PMA and sex in those patients with good cognitive outcomes (see Table 5 and Fig. 4). TBV can be accurately estimated using 2D US measurements of three orthogonal axes (biparietal diameter, vertical axis, and anteroposterior axis) as we have previously shown [11].

**Table 5:** TBV percentiles (cm^3^) by postmenstrual age in preterm infants with normal 2-year cognitive outcome

	**Males**					**Females**				
GA	**p5**	**p10**	**p50**	**p90**	**p95**	**p5**	**p10**	**p50**	**p90**	**p95**
25	80.34	88.64	99.79	111.77	125.18	87.24	88.12	102.23	104.55	107.92
26	91.87	100.78	116.33	132.69	145.83	95.91	97.96	117.38	122.88	126.36
27	103.39	112.92	132.86	153.62	166.48	104.57	107.8	130.93	141.2	144.81
28	114.91	125.05	149.39	174.54	187.13	113.24	117.64	144.48	159.52	163.25
29	126.43	137.19	165.93	195.46	207.79	121.9	127.48	158.03	177.84	181.7
30	137.96	149.32	182.46	216.38	228.44	130.57	137.32	171.58	196.17	200.14
31	149.48	161.46	199	237.3	249.09	139.23	147.16	185.13	214.49	218.59
32	161	173.59	215.53	258.23	269.74	147.9	157	198.68	232.81	237.03
33	172.52	185.73	232.07	279.15	290.39	156.56	166.83	212.24	251.13	255.48
34	184.04	197.87	248.6	300.07	311.04	165.23	176.68	225.79	269.45	273.93
35	195.57	210	265.13	321	331.69	173.89	186.52	239.34	287.77	292.37
36	207.09	222.14	281.67	341.92	352.35	182.56	196.36	252.89	306.1	310.82
37	218.61	234.27	298.2	362.84	373	191.89	206.2	266.44	324.42	329.26
38	230.13	246.41	314.74	383.76	393.65	199.89	216.03	279.99	342.74	347.71
39	241.66	258.54	331.27	404.69	414.3	208.55	225.87	293.54	361.06	366.15
40	253.18	270.68	347.81	425.61	434.95	217.21	235.71	307.09	379.38	384.6

A detailed table of mean TBV by PMA and the population 95% confidence interval by PMA and sex is shown on Tables S4 in supplemental material.

## Discussion

The study of TBV through serial US during NICU admission of VLBWI has allowed us to identify an early deviated pattern of brain growth related to 2-year neurodevelopmental outcome. While we have previously shown that TBV can be monitored accurately through 3D and 2D US [11] and we have shown that those preterm infants born at lower gestational ages and exposed to a greater number of comorbidities had smaller brain volumes related to moderate/severe findings in term-equivalent MRI, this study adds insights into the usefulness of early brain growth monitoring [12].

We found TBV was independently associated with the 2-year neurodevelopmental outcome, with those having good cognitive outcomes showing greater brain volume and brain growth during early postnatal life. In line with other studies, good cognitive outcome was also associated with GA at birth, being female, and the maternal level of education; being SGA, and having moderate to severe MRI findings had a negative impact on the 2-year cognitive outcome [14–18].

Similarly, TBV evolution during early postnatal life was associated with language scores in our preterm study population. Nevertheless, other variables were more importantly related to language outcome, with TBV losing significance when studied with such variables. Thus, language outcome showed a positive association with GA at birth and maternal level of education, while those children with moderate or severe brain injury had lower language scores at two years of age as shown elsewhere [19–23].

In turn, we found no association of TBV and brain growth during the first postnatal weeks and later motor scores at 2 years. We did find that motor outcome was affected by GA at birth, sex (with better motor scores in females), being SGA, and the presence of moderate to severe brain injury on term-MRI, as also supported by previous evidence in the literature [16, 24–27].

We have estimated the population mean of TBV per week of PMA and calculated TBV centiles in those preterm with good cognitive outcome to assist clinicians with normative reference values. This could help incorporating routine TBV monitoring during NICU admission.

The relationship between structural alterations on MRI at term corrected age and adverse long-term neurodevelopmental outcome has been extensively studied [28–30]. Some studies have related total and regional brain volumes measured on MRI at term to motor, cognitive, language, executive, and behavioral functioning in childhood [31–35]. Soria et al. [36] found reduced regional white and gray matter volumes and decreased intellectual functioning in their cohort of low-risk preterm newborns. Arhan et al. [37], similarly, studied regional brain volumes in low-risk preterm infants, identifying smaller volumes than their term controls, with these smaller regional volumes being associated with worse cognitive scores. Bolk et al. [38] found a positive association of volumes in specific brain areas, fine motor skills and visuomotor integration. Kelly et al. [39] found a relationship between white and gray matter volumes with cognitive and language outcomes, with no differences found in motor or behavioral scores.

Few studies have explored brain volumes earlier than term corrected age and through US, as we have seen, volume segmentations have been mostly performed at term equivalent MRI. In recent years, some authors have been interested in investigating early brain volume and their possible association with short- and long-term neurological prognosis. Graça et al. [40] studied a cohort of 128 infants (72 very preterm infants at term equivalent age and 56 term infants during their first postnatal week) in which they estimated brain volumes from intracranial diameters measured on brain US at term, finding that, even in the absence of structural brain damage or major cerebral lesions, preterm infants had smaller brain volumes than term infants. Similarly, they found that smaller brain volumes at term were associated with lower GA at birth, lower birth weight and being SGA. While they were one of the first research groups to study brain volume from ultrasound images, they did not perform early brain volume estimation, nor did they recruit a longitudinal cohort as these preterm infants were assessed after term corrected age. Moreover, in contrast to our previous report [11], their model was not validated in relation to manual segmentation or MRI based TBV estimation. Simsek et al. [41] developed a similar model of estimating brain volume from intracranial diameters measured on ultrasound images based on an ellipsoid, studying brain volume longitudinally in a cohort of 121 preterm infants from the first postnatal days until 34 weeks of corrected age. Subsequently, they related lower brain volumes to poorer neurodevelopment outcomes assessed at two years of age. This was one of the first studies to investigate the relationship between brain volumes measured by US and neurodevelopment in VLBW preterm infants, although it was also based on indirect measurements of brain volume. Furthermore, Cuzzilla et al. [42] evaluated brain growth using sequential cUS regional linear measures from birth to term-equivalent age in a cohort of 139 infants born at < 30 weeks and related it to cognitive, language, and motor outcome at two years of age. They found a positive relationship between the growth of the corpus callosum, cerebellum, and vermis with cognitive and language scores; in contrast, no relationship was demonstrated between tissue measurements and motor scores. This study, unlike the previous ones, did not estimate total brain volume, but instead assessed brain growth through a series of multiple linear measurements at different levels of brain tissue, directly studying the relationship of these isolated measurements with the prognosis at two years. Our study provides new insights into the study of brain growth, thanks to the use of 3D ultrasound, directly assessing total brain volume sequentially from the time of birth, and subsequently relating it to long-term neurological prognosis.

Our study suggests that we can identify an early deviation of the trajectory of brain growth in those preterm infants who will have worse cognitive scores in the long term. We have established reference values that would enable the clinician to identify preterm infants who, despite not necessarily showing brain injury, have an altered brain growth pattern and, therefore, are at a higher risk of presenting adverse neurodevelopmental outcomes.

This study has some limitations that should be acknowledged. Firstly, we had a small number of patients who had adverse neurological outcomes, which has limited us from more robust statistical analysis. Regional volumes may further explain the relationship between early brain growth and neurodevelopmental outcome. We measured TBV and not regional brain volume, which is warranted in future research.

## Conclusions

Measurement of smaller TBV by serial ultrasound during the first weeks of life and up to term age is associated with poor cognitive prognosis at two years of age. Using a sequence of ultrasound scans, we can detect a deviation of brain growth in patients who will have worse cognitive outcomes. We propose normal values for TBV that can serve as a reference as part of the overall assessment in NICU incubators.

### Supplementary information

Below is the link to the electronic supplementary material.

Supplementary file1 (DOCX 2873 KB)

## Data Availability

The data from this study are available from the corresponding author upon reasonable request.

## Abbreviations

BPD: Bronchopulmonary dysplasia
CMV: Cytomegalovirus
GA: Gestational age
IVH: Intraventricular hemorrhage
MRI: Magnetic resonance imaging
NICU: Neonatal intensive care unit
PHVD: Posthemorrhagic ventricular dilatation
PMA: Postmenstrual age
ROP: Retinopathy of prematurity
SES: Socioeconomic status
SGA: Small for gestational age
TBV: Total brain volume
US: Ultrasound
VLBWI: Very low birth weight infants
VOCAL: Virtual Organ Computer-Aided Analysis
WMI: White matter injury

## References

[1] Ream MA, Lehwald L (2018). Neurologic consequences of preterm birth. Curr Neurol Neurosci Rep.

[2] Patel RM (2016). Short- and long-term outcomes for extremely preterm infants. Am J Perinatol.

[3] Moore T, Hennessy EM, Myles J, Johnson SJ, Draper ES, Costeloe KL, Marlow N (2012). Neurological and developmental outcome in extremely preterm children born in England in 1995 and 2006: the EPICure studies. BMJ.

[4] Pierrat V, Marchand-Martin L, Arnaud C, Kaminski M, Resche-Rigon M, Lebeaux C, Bodeau-Livinec F, Morgan AS, Goffinet F, Marret S, Ancel PY, group E-w,  (2017). Neurodevelopmental outcome at 2 years for preterm children born at 22 to 34 weeks’ gestation in France in 2011: EPIPAGE-2 cohort study. BMJ.

[5] Cao Y, Jiang S, Sun J, Hei M, Wang L, Zhang H, Ma X, Wu H, Li X, Sun H, Zhou W, Shi Y, Wang Y, Gu X, Yang T, Lu Y, Du L, Chen C, Lee SK, Zhou W, Chinese Neonatal N (2021). Assessment of neonatal intensive care unit practices, morbidity, and mortality among very preterm infants in China. JAMA Netw Open.

[6] Serenius F, Källén K, Blennow M, Ewald U, Fellman V, Holmström G, Lindberg E, Lundqvist P, Maršál K, Norman M, Olhager E, Stigson L, Stjernqvist K, Vollmer B, Strömberg B, Group E (2013). Neurodevelopmental outcome in extremely preterm infants at 2.5 years after active perinatal care in Sweden. JAMA.

[7] Hirschberger RG, Kuban KCK, O’Shea TM, Joseph RM, Heeren T, Douglass LM, Stafstrom CE, Jara H, Frazier JA, Hirtz D, Rollins JV, Paneth N, Investigators ES (2018). Co-occurrence and severity of neurodevelopmental burden (cognitive impairment, cerebral palsy, autism spectrum disorder, and epilepsy) at age ten years in children born extremely preterm. Pediatr Neurol.

[8] García P, San Feliciano L, Benito F, García R, Guzmán J, Salas S, Fernández C, Prado Del N, Ciprián D, Figueras J (2013) SEN1500 hpa, 2013 Evolución a los 2 años de edad corregida de una cohorte de recién nacidos con peso inferior o igual a 1.500 g de los hospitales pertenecientes a la red neonatal SEN1500 [Outcome at two years corrected age of a cohort of very low birth weight infants from hospitals within the neonatal SEN1500 network] An Pediatr (Barc) 79:279–287 10.1016/j.anpedi.2013.03.017 23684170

[9] Duncan AF, Matthews MA (2018). Neurodevelopmental outcomes in early childhood. Clin Perinatol.

[10] Synnes A, Hicks M (2018). Neurodevelopmental outcomes of preterm children at school age and beyond. Clin Perinatol.

[11] Benavente-Fernández I, Ruiz-González E, Lubian-Gutiérrez M, Lubián-Fernández SP, Cabrales Fontela Y, Roca-Cornejo C, Olmo-Duran P, Lubián-López SP (2021) Ultrasonographic estimation of total brain volume: 3D reliability and 2D estimation. Enabling Routine Estimation During NICU Admission in the Preterm Infant. Front Pediatr 9:708396 10.3389/fped.2021.708396 PMC8339409 34368031

[12] Ruiz-Gonzalez E, Benavente-Fernandez I, Lubian-Gutierrez M, Segado-Arenas A, Zafra-Rodriguez P, Mendez-Abad P, Lubian-Lopez SP (2023) Ultrasonographic evaluation of the early brain growth pattern in very low birth weight infants. Pediatr Res 10.1038/s41390-022-02425-w 36624287

[13] Kidokoro H, Neil JJ, Inder TE (2013). New MR imaging assessment tool to define brain abnormalities in very preterm infants at term. AJNR Am J Neuroradiol.

[14] Linsell L, Malouf R, Morris J, Kurinczuk JJ, Marlow N (2015). Prognostic factors for poor cognitive development in children born very preterm or with very low birth weight: a systematic review. JAMA Pediatr.

[15] Kidokoro H, Anderson PJ, Doyle LW, Woodward LJ, Neil JJ, Inder TE (2014). Brain injury and altered brain growth in preterm infants: predictors and prognosis. Pediatrics.

[16] Li SJ, Tsao PN, Tu YK, Hsieh WS, Yao NJ, Wu YT, Jeng SF (2022). Cognitive and motor development in preterm children from 6 to 36 months of age: trajectories, risk factors and predictability. Early Hum Dev.

[17] Eves R, Mendonca M, Baumann N, Ni Y, Darlow BA, Horwood J, Woodward LJ, Doyle LW, Cheong J, Anderson PJ, Bartmann P, Marlow N, Johnson S, Kajantie E, Hovi P, Nosarti C, Indredavik MS, Evensen KI, Raikkonen K, Heinonen K, Zeitlin J, Wolke D (2021). Association of very preterm birth or very low birth weight with intelligence in adulthood: an individual participant data meta-analysis. JAMA Pediatr.

[18] Eryigit Madzwamuse S, Baumann N, Jaekel J, Bartmann P, Wolke D (2015). Neuro-cognitive performance of very preterm or very low birth weight adults at 26 years. J Child Psychol Psychiatry.

[19] Taskila HL, Heikkinen M, Yliherva A, Valimaa T, Hallman M, Kaukola T, Kallankari H (2022). Antenatal and neonatal risk factors in very preterm children were associated with language difficulties at 9 years of age. Acta Paediatr.

[20] van Noort-van der Spek IL, Franken MC, Weisglas-Kuperus N,  (2012). Language functions in preterm-born children: a systematic review and meta-analysis. Pediatrics.

[21] Kovachy VN, Adams JN, Tamaresis JS, Feldman HM (2015). Reading abilities in school-aged preterm children: a review and meta-analysis. Dev Med Child Neurol.

[22] Sentenac M, Johnson S, Charkaluk ML, Seppanen AV, Aden U, Cuttini M, Maier R, Mannamaa M, Zeitlin J, the Eg,  (2020). Maternal education and language development at 2 years corrected age in children born very preterm: results from a European population-based cohort study. J Epidemiol Community Health.

[23] Ko G, Shah P, Lee SK, Asztalos E (2013). Impact of maternal education on cognitive and language scores at 18 to 24 months among extremely preterm neonates. Am J Perinatol.

[24] Linsell L, Malouf R, Morris J, Kurinczuk JJ, Marlow N (2016). Prognostic factors for cerebral palsy and motor impairment in children born very preterm or very low birthweight: a systematic review. Dev Med Child Neurol.

[25] Kato T, Mandai T, Iwatani S, Koda T, Nagasaka M, Fujita K, Kurokawa D, Yamana K, Nishida K, Taniguchi-Ikeda M, Tanimura K, Deguchi M, Yamada H, Iijima K, Morioka I (2016). Extremely preterm infants small for gestational age are at risk for motor impairment at 3 years corrected age. Brain Dev.

[26] El Rafei R, Jarreau PH, Norman M, Maier RF, Barros H, Van Reempts P, Pedersen P, Cuttini M, Costa R, Zemlin M, Draper ES, Zeitlin J, Group ER (2021). Association between postnatal growth and neurodevelopmental impairment by sex at 2 years of corrected age in a multi-national cohort of very preterm children. Clin Nutr.

[27] Arulkumaran S, Tusor N, Chew A, Falconer S, Kennea N, Nongena P, Hajnal JV, Counsell SJ, Rutherford MA, Edwards AD (2020). MRI findings at term-corrected age and neurodevelopmental outcomes in a large cohort of very preterm infants. AJNR Am J Neuroradiol.

[28] Brouwer MJ, Kersbergen KJ, van Kooij BJM, Benders M, van Haastert IC, Koopman-Esseboom C, Neil JJ, de Vries LS, Kidokoro H, Inder TE, Groenendaal F (2017). Preterm brain injury on term-equivalent age MRI in relation to perinatal factors and neurodevelopmental outcome at two years. PLoS ONE.

[29] Woodward LJ, Anderson PJ, Austin NC, Howard K, Inder TE (2006). Neonatal MRI to predict neurodevelopmental outcomes in preterm infants. N Engl J Med.

[30] Van ’t Hooft J, van der Lee JH, Opmeer BC, Aarnoudse-Moens CS, Leenders AG, Mol BW, de Haan TR,  (2015). Predicting developmental outcomes in premature infants by term equivalent MRI: systematic review and meta-analysis. Syst Rev.

[31] Cheong JL, Thompson DK, Spittle AJ, Potter CR, Walsh JM, Burnett AC, Lee KJ, Chen J, Beare R, Matthews LG, Hunt RW, Anderson PJ, Doyle LW (2016). Brain volumes at term-equivalent age are associated with 2-year neurodevelopment in moderate and late preterm children. J Pediatr.

[32] Keunen K, Isgum I, van Kooij BJ, Anbeek P, van Haastert IC, Koopman-Esseboom C, Fieret-van Stam PC, Nievelstein RA, Viergever MA, de Vries LS, Groenendaal F, Benders MJ (2016). Brain volumes at term-equivalent age in preterm infants: imaging biomarkers for neurodevelopmental outcome through early school age. J Pediatr.

[33] Katusic A, Raguz M, Zunic Isasegi I (2020) Brain tissue volumes at term-equivalent age are associated with early motor behavior in very preterm infants. Int J Dev Neurosci 10.1002/jdn.10039 32433785

[34] Lind A, Haataja L, Rautava L, Valiaho A, Lehtonen L, Lapinleimu H, Parkkola R, Korkman M, Group PS (2010). Relations between brain volumes, neuropsychological assessment and parental questionnaire in prematurely born children. Eur Child Adolesc Psychiatry.

[35] Liverani MC, Loukas S, Gui L, Pittet MP, Pereira M, Truttmann AC, Brunner P, Bickle-Graz M, Huppi PS, Meskaldji DE, Borradori-Tolsa C (2023). Behavioral outcome of very preterm children at 5 years of age: prognostic utility of brain tissue volumes at term-equivalent-age, perinatal, and environmental factors. Brain Behav.

[36] Soria-Pastor S, Padilla N, Zubiaurre-Elorza L, Ibarretxe-Bilbao N, Botet F, Costas-Moragas C, Falcon C, Bargallo N, Mercader JM, Junque C (2009). Decreased regional brain volume and cognitive impairment in preterm children at low risk. Pediatrics.

[37] Arhan E, Gucuyener K, Soysal S, Salvarli S, Gurses MA, Serdaroglu A, Demir E, Ergenekon E, Turkyilmaz C, Onal E, Koc E, Atalay Y (2017). Regional brain volume reduction and cognitive outcomes in preterm children at low risk at 9 years of age. Childs Nerv Syst.

[38] Bolk J, Padilla N, Forsman L, Brostrom L, Hellgren K, Aden U (2018). Visual-motor integration and fine motor skills at 6½ years of age and associations with neonatal brain volumes in children born extremely preterm in Sweden: a population-based cohort study. BMJ Open.

[39] Kelly CE, Thompson DK, Spittle AJ, Chen J, Seal ML, Anderson PJ, Doyle LW, Cheong JL (2020). Regional brain volumes, microstructure and neurodevelopment in moderate-late preterm children. Arch Dis Child Fetal Neonatal Ed.

[40] Graca AM, Cardoso KR, da Costa JM, Cowan FM (2013). Cerebral volume at term age: comparison between preterm and term-born infants using cranial ultrasound. Early Hum Dev.

[41] Simsek GK, Canpolat FE, Buyuktiryaki M, Okman E, Keser M, Ustunyurt Z, Kutman HGK (2020). Developmental outcomes of very low birthweight infants with non-hemorrhagic ventricular dilatations and the relationships thereof with absolute brain volumes measured via two-dimensional ultrasonography. Childs Nerv Syst.

[42] Cuzzilla R, Cowan FM, Rogerson S, Anderson PJ, Doyle LW, Cheong JLY, Spittle A (2023) Relationships between early postnatal cranial ultrasonography linear measures and neurodevelopment at 2 years in infants born at <30 weeks’ gestational age without major brain injury. Arch Dis Child Fetal Neonatal Ed 10.1136/archdischild-2022-324660 36958812

