# Supplementary material for: Relationship of early brain growth pattern measured by ultrasound with neurological outcome at two years of age in very low birth weight infants
Source: Eur J Pediatr. 2023 Sep 8;182(11):5119–29. doi: 10.1007/s00431-023-05170-2 (PMC10640451; doi:10.1007/s00431-023-05170-2)
Supplement: Supplementary file 1 — Supplementary file1 (DOCX 2873 KB) [file 431_2023_5170_MOESM1_ESM.docx]

| **VARIABLES** | |
| --- | --- |
| **Small for gestational age (SGA)** | Those with birth weight bellow the 10th percentile for gestational age. |
| **Socioeconomic status (SES)** | SES was measured related to the level of maternal education and classified in three categories: low (primary and secondary education), medium (high school studies and professional training modules) and high (university and postgraduate studies). |
| **Late onset sepsis** | Clinical condition consisting of systemic signs of infection and isolation of a bacterial pathogen in blood culture after the first 72 hours of life. |
| **Significant patent ductus arteriosus (PDA)** | PDA requiring surgical or pharmacological closure. |
| **Severe retinopathy of prematurity (ROP)** | Retinal vasculopathy stage 3 or higher^[1].^ |
| **Necrotizing enterocolitis (NEC)** | Confirmed necrotizing enterocolitis (Bell Stage II or higher)^[2].^ |
| **Moderate to severe bronchopulmonary dysplasia (BPD)** | Need for supplemental oxygen and/or positive pressure at 36 weeks postmenstrual age or at discharge among VLBWI born < 32 weeks and at 56 days postnatal age or at discharge in VLBWI born >32 weeks^[3].^ |
| **Total days of respiratory support** | Duration of invasive and non-invasive respiratory support in days. |
| **Duration of parenteral nutrition (TPN)** | Total days of TPN received. |
| **Pathological US** | Ultrasonography with any abnormal finding in US: grade I-II-III of GM-IVH, PHI, any degree of WMI^[4].^ |
| **Severe brain injury** | Grade 3 GM-IVH, PHI and/or moderate to severe WMI^[5].^ |

**Table s1.** Definition of perinatal and postnatal variables.

*1. International Committee for the Classification of Retinopathy of P (2005) The International Classification of Retinopathy of Prematurity revisited. Arch Ophthalmol 123:991-999*

*2. Bell MJ, Ternberg JL, Feigin RD, Keating JP, Marshall R, Barton L, Brotherton T (1978) Neonatal necrotizing enterocolitis. Therapeutic decisions based upon clinical staging. Ann Surg 187:1-7*

*3. Bancalari E, Claure N (2006) Definitions and diagnostic criteria for bronchopulmonary dysplasia. Semin Perinatol 30:164-170*

*4. Agut T, Alarcon A, Cabanas F, Bartocci M, Martinez-Biarge M, Horsch S, eur USbg (2020) Preterm white matter injury: ultrasound diagnosis and classification. Pediatr Res 87:37-49*

*5. Volpe JJ (1981) Neurology of the newborn. Major Probl Clin Pediatr 22:1-648*

|  | | **MOTOR OUTCOME** | | | **COGNITIVE OUTCOME** | | | **LANGUAGE OUTCOME** | | | **TOTAL** |
| --- | --- | --- | --- | --- | --- | --- | --- | --- | --- | --- | --- |
|  | | **Adverse (n=13)** | **Favorable (n=92)** | **p** | **Adverse (n=9)** | **Favorable (n=96)** | **p** | **Adverse (n=22)** | **Favorable (n=83)** | **p** | **Total (n=105)** |
| **Gestational age (weeks)** | | 29,6 (± 2,1) | 29,3 (± 2,3) | 0.83 | 27,7 (± 3,1) | 29,4 (± 2,1) | 0.094 | 29 (± 2,4) | 29,4 (± 2,2) | 0.518 | 29,3 (± 2,3) |
| **Sex (F)** | | 4 (30,77%) | 49 (53,26%) | 0.15 | 2 (22,22%) | 51 (53,13%) | 0.093 | **6 (27,27%)** | **47 (56,63%)** | **0.017** | 53 (50,48%) |
| **SGA** | | 3 (23,08%) | 14 (15,22%) | 0.44 | 2 (22,22%) | 15 (15,63%) | 0.636 | 6 (27,27%) | 11 (13,25%) | 0.188 | 17 (16,19%) |
| **Birth weight (grams)** | | 1240,8 (± 362,7) | 1158,4 (± 364) | 0.468 | 1085 (± 531,1) | 1176,4 (± 346,2) | 0.492 | 1117,7 (± 416,2) | 1182 (± 349,2) | 0.447 | 1168,6 (± 363,1) |
| **Birth head circumference (cm)** | | 29,3 (± 2,9) | 27,4 (± 3) | 0.104 | 28,7 (± 4,3) | 27,6 (± 2,9) | 0.221 | 27,9 (± 3,4) | 27,6 (± 3) | 0.649 | 27,7 (± 3,1) |
| **Multiple birth** | | 7 (53,85%) | 34 (36,96%) | 0.363 | **7 (77,78%)** | **34 (35,42%)** | **0.026** | 9 (40,91%) | 32 (38,55%) | 1 | 41 (39,05%) |
| **Prenatal steroids** | | **7 (53,85%)** | **75/88 (85,23%)** | **0.015** | 5 (55,56%) | 77/92 (83,7%) | 0.062 | 17 (77,27%) | 65/79 (82,28%) | 0.554 | 82/101 (81,19%) |
| **Clinical chorioamnionitis** | | 1 (7,69%) | 14 (15,12%) | 0.687 | 3 (33,3%) | 12 (12,5%) | 0.117 | 4 (18,18%) | 11 (13,25%) | 0.513 | 15 (14,29%) |
| **Apgar 1 min** | | 6 [4 – 7] | 6 [5 – 7] | 0.739 | 7 [7 – 7] | 6 [5 – 7] | 0.283 | 7 [6 – 7] | 6 [5 – 7] | 0.189 | 6 [5 – 7] |
| **Apgar 5 min** | | 8 [7 – 8] | 8 [7 – 9] | 0.732 | 8 [8 – 9] | 8 [7 – 8] | 0.275 | 8 [7 – 9] | 8 [7 – 8] | 0.34 | 8 [7 – 8] |
| **CRIB index** | | 1 [1 – 3] | 1 [0 – 4] | 0.763 | 1 [0 – 7] | 1 [0 – 4] | 0.59 | 1 [0 – 4] | 1 [0 – 4] | 0.909 | 1 [0 – 4] |
| **Level of**  **maternal education** | **Low** | 10 (76.92%) | 46/88 (52,27%) | 0.204 | 7 (77,78%) | 49/92 (53,26%) | 0.418 | 14/20 (70%) | 42/81 (51,85%) | 0.106 | 56/101 (55,45%) |
|  | **Medium** | 3 (23.08%) | 29/88 (32,95%) |  | 2 (22,22%) | 30/92 (32,61%) |  | 6/20 (30%) | 26/81 (32,10%) |  | 32/101 (31,68%) |
|  | **High** | 0 (0%) | 13/88 (14,77%) |  | 0 (0%) | 13/92 (14,13%) |  | 0 (0%) | 13/81 (16,05%) |  | 13/101 (12,87%) |
| **Early sepsis** | | 0 (0%) | 2 (2,17%) | 1 | 0 (0%) | 2 (2,08%) | 1 | 0 (0%) | 2 (2,41%) | 1 | 2 (1,9%) |
| **Late sepsis** | | 5 (38,46%) | 22 (23,91%) | 0.312 | 3 (33,3%) | 24 (25%) | 0.691 | 5 (22,73%) | 22 (26,51%) | 0.791 | 27 (25,71%) |
| **Significant PDA** | | 4 (30,77%) | 9 (9.78%) | 0.054 | 2 (22,22%) | 11 (11,46%) | 0.308 | 4 (18,18%) | 9 (10,84%) | 0.465 | 13 (12,38%) |
| **Severe ROP** | | **4 (30,77%)** | **3/91 (3,3%)** | **0.004** | 2 (22,2%) | 5/95 (5,26%) | 0.112 | **4 (18,88%)** | **3/82 (3,66%)** | **0.035** | 7 /104 (6,73%) |
| **Confirmed NEC** | | 0 (0%) | 1 (1,09%) | 1 | 0 (0%) | 1 (1,04%) | 1 | 1 (4.55%) | 0 (0%) | 0.21 | 1 (0,95%) |
| **Mod/severe BPD** | | **6 (46,15%)** | **13/90 (14.44%)** | **0.014** | 3 (33,3%) | 16/94 (17,02%) | 0.361 | 7 (31,82%) | 12/81 (14,81%) | 0.117 | 19/103 (18,45%) |
| **Total respiratory support (days)** | | 25 [14 – 81] | 14 [5 – 55] | 0.075 | 68 [14 – 90] | 15 [5 – 55] | 0.096 | 17 [6 – 81] | 15 [5 – 47] | 0.275 | 15 [5.5 – 60] |
| **TPN (days)** | | 17 [13 – 23] | 12 [7 – 21] | 0.079 | 18 [15 – 42] | 13 [7 – 20] | 0.068 | 18 [9 – 25] | 13 [7 – 19] | 0.076 | 13.5 [7.5 – 21] |
| **Pathological US** | | 5 (38,46%) | 18 (19,57%) | 0.153 | 3 (33,3%) | 20 (20,83%) | 0.407 | 6 (27,27%) | 17 (20,48%) | 0.564 | 23 (21,9%) |
| **IVH 3** | | **3 (23,08%)** | **3 (3,26%)** | **0.024** | 1 (11,1%) | 5 (5,21%) | 0.424 | 2 (9,09%) | 4 (4,82%) | 0.603 | 6 (5,71%) |
| **PHI** | | 1 (7,69%) | 2 (2,17%) | 0,33 | 1 (11,1%) | 2 (2,08%) | 0.238 | 2 (9,09%) | 1 (1,2%) | 0.11 | 3 (2,86%) |
| **Mod/severe WMI** | | **2 (15,38%)** | **0 (0%)** | **0.014** | 1 (11,1%) | 1 (1,04%) | 0.165 | **2 (9,09%)** | **0 (0%)** | **0.042** | 2 (1,9%) |
| **Kidokoro** | | **2 [0-10]** | **2 [0-2]** | **0.03** | 1 [0-12] | 2 [0-2] | 0.13 | 1 [0-9] | 2 [0-2] | 0.07 | 2 [0-3] |
| **Sum of comorbidities** | | **1 [0-3]** | **0 [0-1]** | **0.006** | 1 [0 – 1] | 0 [0 – 1] | 0.255 | 0 [0 - 2] | 0 [0 – 1] | 0.477 | 0 [0 – 1] |

**Table s2.** Clinical characteristics related to 2-year neurodevelopmental outcomes.

*SGA: Small for gestational age. PDA: Patent ductus arteriosus. ROP: Retinopathy of prematurity. NEC: necrotizing enterocolitis. BPD: bronchopulmonary dysplasia. TPN: Parenteral nutrition. Pathological US: Ultrasonography with any abnormal finding in US. IVH: Intraventricular hemorrhage. PHI: parenchymal hemorrhagic infarction. WMI: white matter injury. TBV: Total brain volume.*

|  | **GLOBAL** | **GOOD COGNITIVE OUTCOME** | **ADVERSE COGNITIVE OUTCOME** |
| --- | --- | --- | --- |
| **Const** | 18.31 | 18.78 | 13.83 |
| **Coef33^1^** | 0.33 | 0.23 | 0.64 |
|  | Nº obs = 539 / Nº groups = 104  Wald chi2 = 5,26 / P model = 0,0218 | Nº obs = 493 / Nº groups = 95  Wald chi2 = 2,23 / P model = 0,1354 | Nº obs = 46 / Nº groups = 9  Wald chi2 = 5,10 / P model = 0,0239 |

**Table s3.** Brain growth rate related to cognitive outcome.

*^1^PMA is centered at 33 weeks to facilitate the interpretability of the constant.*

| **PMA (weeks)** | **Males** | | **Females** | |
| --- | --- | --- | --- | --- |
|  | **Mean** | **95% CI for population means** | **Mean** | **95% CI for population means** |
| **25** | 98.24 | 95.37-105.91 | 90.59 | 81.04-101.92 |
| **26** | 109.41 | 107.87-131.6 | 106.88 | 97.71-114.1 |
| **27** | 126.57 | 114.61-145.17 | 123.18 | 108.7-127.92 |
| **28** | 148.74 | 145.54-171.73 | 139.47 | 126.51-142.99 |
| **29** | 160.89 | 154.89-177.05 | 155.76 | 153.35-170.68 |
| **30** | 188.06 | 181.59-202.66 | 172.05 | 166.24-182.91 |
| **31** | 195.23 | 184.11-201.02 | 188.35 | 179.42-195.99 |
| **32** | 212.39 | 200.2-218.42 | 204.64 | 195.56-214.57 |
| **33** | 229.56 | 214.4-237.56 | 220.93 | 211.34-228.39 |
| **34** | 246.72 | 224.44-253.14 | 237.22 | 220.69-245.02 |
| **35** | 263.89 | 261.83-294.56 | 253.52 | 240.54-267.41 |
| **36** | 281.05 | 262.91-302.6 | 269.81 | 253.32-286.88 |
| **37** | 298.21 | 262.92-316.81 | 276.10 | 235.32-284.26 |
| **38** | 315.38 | 292.87-343.85 | 302.39 | 241.22-307.18 |
| **39** | 332.54 | 281.6-396.83 | 318.69 | 250.12-341.06 |
| **40** | 349.71 | 308.61-406.91 | 334.98 | 219.94-373.38 |

**Table s4.** Study population means and 95% confidence interval for population means of US TBV (cm3) by post-menstrual age in preterm infants with normal 2 year cognitive outcome.

**

**

***Figure s1.*** *Flow diagram of patients included.*
